# SARS-CoV-2 Infection, Vaccination, and Irritable Bowel Syndrome in Veterans: A Cross-Sectional Study

**DOI:** 10.11648/j.ijg.20240802.14

**Published:** 2024-11-29

**Authors:** Arash Oliver Parsi, George Nicholas Verne, Yu Jiang, Sue Ann Theus, Josh Sullivan, Qiqi Zhou

**Affiliations:** 1 Metairie Park Country Day School, Metairie, USA; 2 College of Medicine, University of Tennessee, Memphis, USA; 3 Lt. Col. Luke Weathers, Jr. VA Medical Center, Memphis, USA; 4 Division of Epidemiology, Biostatistics, and Environmental Health, School of Public Health, University of Memphis, Memphis, USA

**Keywords:** Coronavirus Infection, COVID, Irritable Bowel Syndrome, IBS, Vaccination

## Abstract

**Background:**

The association and interaction between SARS-CoV-2 (COVID-19) infection and irritable bowel syndrome (IBS) has not been adequately explored. We sought to determine whether a mechanistic relationship exists and whether vaccination against COVID-19 plays a role in this relationship.

**Methods:**

Using the Veterans Affairs (VA) electronic healthcare database, we obtained a random sample of veterans in October 2022 for this cross-sectional study. Demographic characteristics of the participants (e.g., age, sex, race), prior COVID infection, COVID vaccination status, and IBS diagnosis were extracted from the VA medical records. Univariate and multivariate logistic regression analyses were performed to determine potential associations between the listed factors and IBS diagnosis.

**Results:**

A total of 9,112 veterans were analyzed. In univariate analysis, race, COVID infection, and vaccination were significantly associated with IBS. In multivariate regression analysis, only the effects of COVID infection and vaccination were statistically significant. The odds ratios for development of IBS among veterans with COVID infection versus those without was 1.94 (95% CI: 1.53–2.45), and for vaccinated versus unvaccinated, was 1.49 (95% CI: 1.18–1.89). Further analysis showed that vaccinated veterans who did not contract COVID infection had a lower risk of developing IBS compared to unvaccinated veterans who contracted COVID.

**Conclusions:**

The results of this study suggest that veterans with COVID infection are almost twice as likely to develop IBS compared to those who have never had COVID infection. Vaccinated veterans have a lower risk of contracting COVID and subsequently, a diminished chance of developing IBS. Overall, vaccination of veterans with protection against COVID diminishes the risk of IBS development.

## 1. Introduction

The COVID pandemic has had a significant impact on global health, with hundreds of millions of cases and deaths reported worldwide [1]. According to the Centers for Disease Control and Prevention (CDC), more than 100 million Americans were infected with COVID by May 2023 [2]. The CDC also estimates that approximately 10% of infected individuals develop some form of sequela (long COVID) that could last weeks to months or even longer [3, 4].

While the primary manifestations of COVID infection are respiratory in nature, the virus may affect other organ systems, including the gastrointestinal (GI) tract [5, 6]. Some individuals with infection may experience GI symptoms such as diarrhea, nausea, or abdominal pain. It is important to note that respiratory symptoms such as cough and shortness of breath are more commonly associated with COVID-19. However, the presence of GI symptoms adds to the varied clinical presentations of the disease. SARS-CoV-2 has been detected in the stool of infected individuals, suggesting that it can infect cells of the gastrointestinal tract [7]. The exact mechanism by which the virus affects the GI tract remains under investigation. It is crucial that individuals experiencing symptoms consistent with COVID-19, whether respiratory or gastrointestinal, seek medical advice. Testing for COVID-19 may be recommended, especially if there is known exposure to the virus or if the symptoms are consistent with the disease.

Many GI and liver disorders are attributed to COVID infection [8–15]. One potential GI complication arising from COVID infection is the onset of irritable bowel syndrome (IBS), a common disorder of gut-brain interactions (DGBI). COVID-19 vaccination has emerged as a pivotal tool for mitigating the risks associated with severe illness, hospitalization, and mortality due to the virus. Although the primary emphasis of COVID-19 vaccination has centered on averting respiratory manifestations and complications, preliminary emerging evidence suggests its potential role in attenuating and/or preventing GI sequalae [16]. These preliminary findings suggest that COVID-19 vaccines not only shield against severe respiratory outcomes, but may also diminish the incidence of various symptoms, including those affecting the GI tract. Moreover, vaccinated individuals who contract COVID-19 are predisposed to mild symptoms. It is crucial to acknowledge that breakthrough infections can occur, albeit less frequently, in vaccinated individuals. Nevertheless, vaccines have consistently exhibited efficacy in preventing severe diseases and their associated complications. Ongoing investigations are delving into the broader implications of COVID-19 vaccination, encompassing its impact on transmission dynamics, duration of immunity, and the manifestation of diverse symptoms. Responses to COVID-19 exhibit considerable inter-individual variability, with factors such as pre-existing health conditions, including IBS, influencing disease outcomes. COVID-19 vaccines are designed to protect against a spectrum of symptoms and severity levels. Notwithstanding reports suggesting a link between COVID infection and IBS, this relationship remains inadequately explored in epidemiological studies, presenting a knowledge gap. Therefore, our current study aimed to elucidate whether COVID infection confers a heightened risk for IBS development and to assess how vaccination against COVID influences this potential risk.

## 2. Methods

### 2.1. Study Population

A random sample of veteran patients from October 2022 was drawn from the extensive electronic healthcare database maintained by the United States Department of Veterans Affairs, renowned for comprehensive patient records. Ethical approval for this research was obtained from Lt. Col. Luke Weathers Jr. VA Medical Center in Memphis, TN, reflects our commitment to rigorous ethical standards. Given the observational nature of our study and the utilization of anonymized data, the Institutional Review Board (IRB) granted a waiver for informed consent, recognizing the minimal risk and impracticality of seeking individual consent in this context.

The demographic characteristics of the veterans, including age, sex, and race, were extracted from electronic medical records. Critical medical information such as COVID infection status, COVID vaccination status, and diagnosis of irritable bowel syndrome (IBS) based on the Rome IV classification, was also retrieved. The confirmation of a positive COVID diagnosis relied on polymerase chain reaction (PCR) test results. Patients were classified as vaccinated if they had received at least one dose of any COVID vaccine. Throughout the study, the primary outcome of interest was the IBS diagnosis.

### 2.2. Statistical Analysis

Demographic data were succinctly summarized and meticulously presented, with categorical variables represented by counts and percentages, whereas continuous variables were expressed as mean ± standard deviation (SD). To identify potential associations between various factors and IBS diagnosis, both univariate and multivariate logistic regression analyses were conducted. Results were expressed as odds ratios (OR) accompanied by 95% confidence intervals (CI), providing insights into the strength and direction of the observed associations. All statistical analyses were performed using the comprehensive SAS software (SAS Institute, Cary, NC, USA), ensuring the robustness and reliability of our findings.

## 3. Results

**Figure 1.**
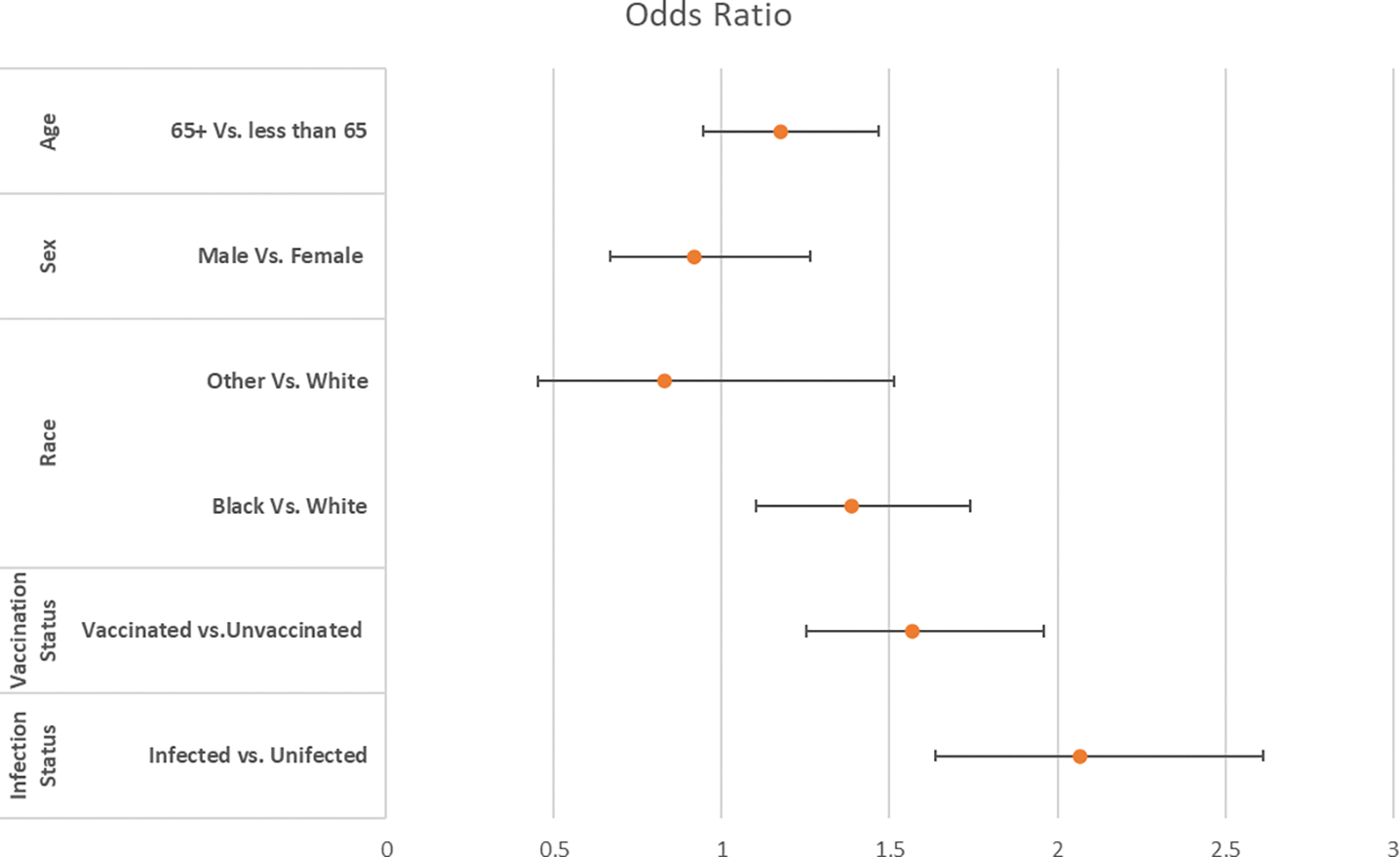
Univariate Analysis Showing the Odds (95% CI) of Developing IBS for Independent Factors.

A sample of 9,112 veterans was analyzed. The baseline characteristics of the study population are summarized in Table 1. The study population consisted of 8,019 male veterans and 1,093 female veterans. A total of 5,038 veterans were vaccinated and 1,651 veterans developed COVID infection. The majority of veterans (4193, 83% of 5038 verterans) received the Pfizer vaccination. Univariate logistic analysis revealed that among the independent variables, race, COVID-19 infection, and COVID vaccination showed statistically significant associations with IBS (Figure 1). 1) Race: According to the univariate analysis, there is a statistically significant association between race and the presence of IBS. The odds of having IBS for black veterans was 1.369 times that of white veterans [odds ratio 1.369, 95% confidence interval (1.100, 1.1704)]. 2) COVID-19 Infection: The analysis indicated a statistically significant association between having a COVID-19 infection and IBS. Veterans who had COVID infection were more likely to have IBS [odds ratio 2.067, 95% confidence interval (1.636, 2.613)]. 3) Similarly, the analysis showed a statistically significant association between receiving a COVID vaccination and the presence of IBS. It is important to note that univariate analysis assesses the relationship between each independent variable and the outcome (in this case, IBS) individually without considering the potential interactions between variables. These findings could have implications in understanding the factors associated with IBS. For example, it might prompt further research to explore the mechanisms or reasons for these associations.

**Figure 2.**
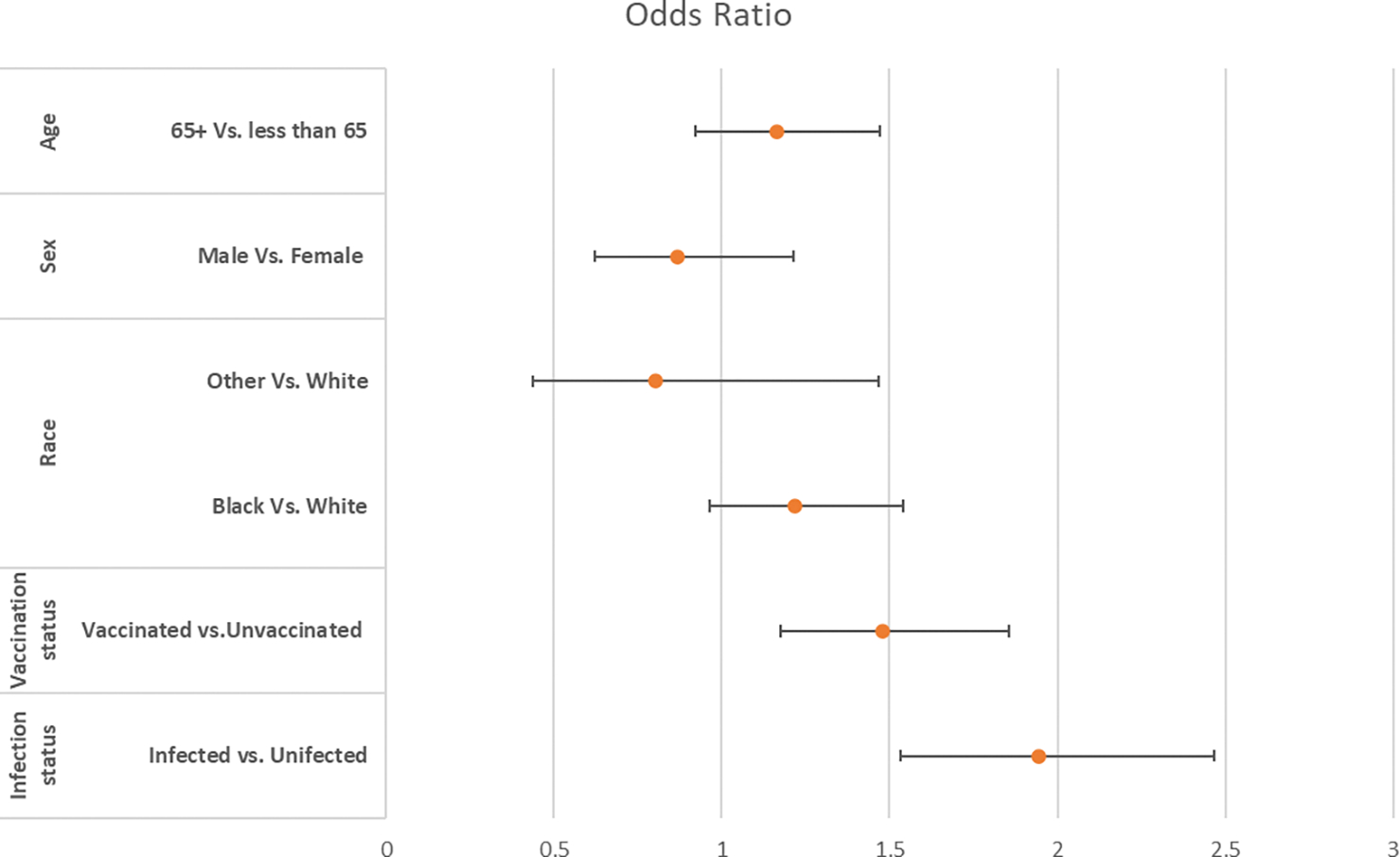
Multivariate Analysis of showing the Odds (95% CI) of Developing IBS for Independent Factors.

Multivariate logistic analysis was also performed to explore the independent contributions of each variable, while accounting for potential confounding factors. Multivariate analysis is a statistical technique that simultaneously analyzes relationships between multiple variables. In this case, we considered the effects of various factors on the presence of IBS, while accounting for the potential influence of other variables. The results of multivariate analysis, especially after adjusting for other factors, provide more nuanced insights into the relationships between variables and irritable bowel syndrome (IBS). Adjusting for other factors means that the analysis considers the potential confounding effects of additional variables. In doing so, the aim is to isolate the specific impact of each variable on the outcome (IBS). In the multivariate analysis, after adjusting for race, sex, and age, the effects of COVID infection and vaccination remained statistically significant (Figure 2). This implies that when considering other variables in the analysis, these two factors (COVID infection and vaccination) still show a statistically significant association with the presence of IBS. The persistence of statistical significance for COVID infection and vaccination, even after adjusting for other factors, suggests that these factors may have an independent association with IBS. This could have implications for understanding the relationship between COVID-related variables and gastrointestinal health.

### 3.1. Effect of COVID Infection

Of the total number of patients, 1,651 were diagnosed with COVID infection. A significantly higher percentage of patients with COVID infection experienced IBS (6.48%, 107/1651) compared to those without COVID infection (3.24%, 242/7461; P< 0.0001). 1) Percentage of Patients with IBS: COVID-19 Group, 6.48% of patients with COVID experienced IBS (107 out of 1651). Non-COVID Group: 3.24% of the patients without COVID infection experienced IBS (242 of 7461). 2) Statistical Significance: The statement mentions that the difference between the two groups is statistically significant, with a p-value of less than 0.0001 (P < 0.0001). The results suggest that a significantly higher percentage of patients with COVID-19 experienced irritable bowel syndrome compared to those without COVID infection. Statistical significance, indicated by the very low p-value (P < 0.0001), suggests that this difference is unlikely to be due to chance alone.

In multivariate logistic regression analysis, after adjusting for age, sex, sex, and covid vaccination, the association of COVID infection with IBS remained statistically significant, with an odds ratio of 1.94 (95% CI: 1.53–2.45; P< 0.001) (Figure 2). This type of statistical analysis is used when multiple independent variables may influence a binary outcome. In this case, the outcome variable is likely whether an individual has COVID infection or not. Multivariate logistic regression allows researchers to control for the influence of other variables often referred to as covariates or confounding factors. By doing this, the analysis aimed to isolate the specific association between COVID infection and IBS, taking into account the potential impact of other relevant variables. The results indicated that even after adjusting for confounding factors, there was a statistically significant association between COVID infection and IBS. The odds ratio is a measure of the strength and direction of the association. In this case, the odds ratio was 1.94, suggesting that individuals with COVID infection have 1.94 times the odds, or are more likely, of having IBS compared to those without COVID infection. The 95% confidence interval provides a range within which we can be reasonably confident that the true odds ratio lies. In this instance, the confidence interval was 1.53 to 2.45. The p-value was less than 0.001 (P < 0.001), indicating that the association was highly statistically significant. This means that there is strong evidence to reject the null hypothesis that there is no association between COVID infection and IBS.

In summary, the study found a significant and positive association between COVID infection and irritable bowel syndrome, after adjusting for other factors, with an odds ratio of 1.94 and a high level of statistical significance.

### 3.2. Effect of COVID Vaccination

In the study population, 5,038 veterans were vaccinated against COVID. A significantly higher percentage of vaccinated veterans had IBS (4.55%, 229/5038) than unvaccinated veterans (2.95%, 120/4074) (P< 0.0001). The analysis suggests that a significantly higher percentage of vaccinated veterans had irritable bowel syndrome than unvaccinated veterans. It is important to note that these results describe an association and that the study design and potential confounding factors should be considered when interpreting the findings.

In multivariate logistic regression analysis, after adjusting for other factors, the association of COVID vaccination with IBS remained statistically significant, with an odds ratio of 1.49 (95%CI: 1.18–1.89); P< 0.001 (Figure 2). Multivariate logistic regression analysis revealed a statistically significant association between COVID vaccination and irritable bowel syndrome in a multivariate logistic regression analysis. The odds ratio of 1.49 suggests an increased odds of IBS among those who received the COVID vaccination after adjusting for other factors. The type of vaccine and number of vaccine doses did not have any effect on the development of IBS. This result is important for interpreting the broader implications of the COVID-19 vaccination in relation to IBS. This suggests that, according to the study findings, the risk of IBS development does not appear to be influenced by the specific type of COVID-19 vaccine administered or the number of doses received.

### 3.3. Combined Effect of COVID Infection and Vaccination

Veterans were stratified into four subgroups based on prior COVID infection and vaccination status. The groups were 1) those without prior COVID infection and without COVID vaccination (C−V−); 2) those without prior COVID infection who had received at least one dose of a vaccine against COVID (C−V+); 3) those with COVID infection who had not received any vaccination against COVID (C+V−); and 4) those with COVID infection who had received at least one dose of a COVID vaccine (C+V+).

The reference group (C−V−) was used as a baseline for comparison, and odds ratios and 95% confidence intervals were calculated for the development of irritable bowel syndrome (IBS) in each of the other three groups relative to the reference group. The odds ratios indicate whether the odds of developing IBS are higher or lower in the other groups than in the C−V− group, and the confidence intervals provide a range within which the true odds ratio is likely to fall. This approach allows researchers to examine how the combination of prior COVID infection and vaccination status may be associated with the development of IBS while controlling for other factors. In multivariate logistic regression analysis, after adjusting for other factors, the C−V+, C+V−, and C+V+ groups all had higher odds ratios (greater than 1) when compared with the C−V− reference group, which were 1.487 (95% CI: 1,135, 1.948), 1.975 (95% CI: 1.31, 2977), and 2.872 (95% CI: 2.071, 3.984), respectively. All of the odds ratios had p-values smaller than 0.05, which were 0.004, 0.0012, and <0.0001 p-values, respectively. Since all three groups have odds ratios significantly larger than 1, it indicates that veterancs in these groups are more likely to have IBS compared to the reference C-V-group.

**Figure 3.**
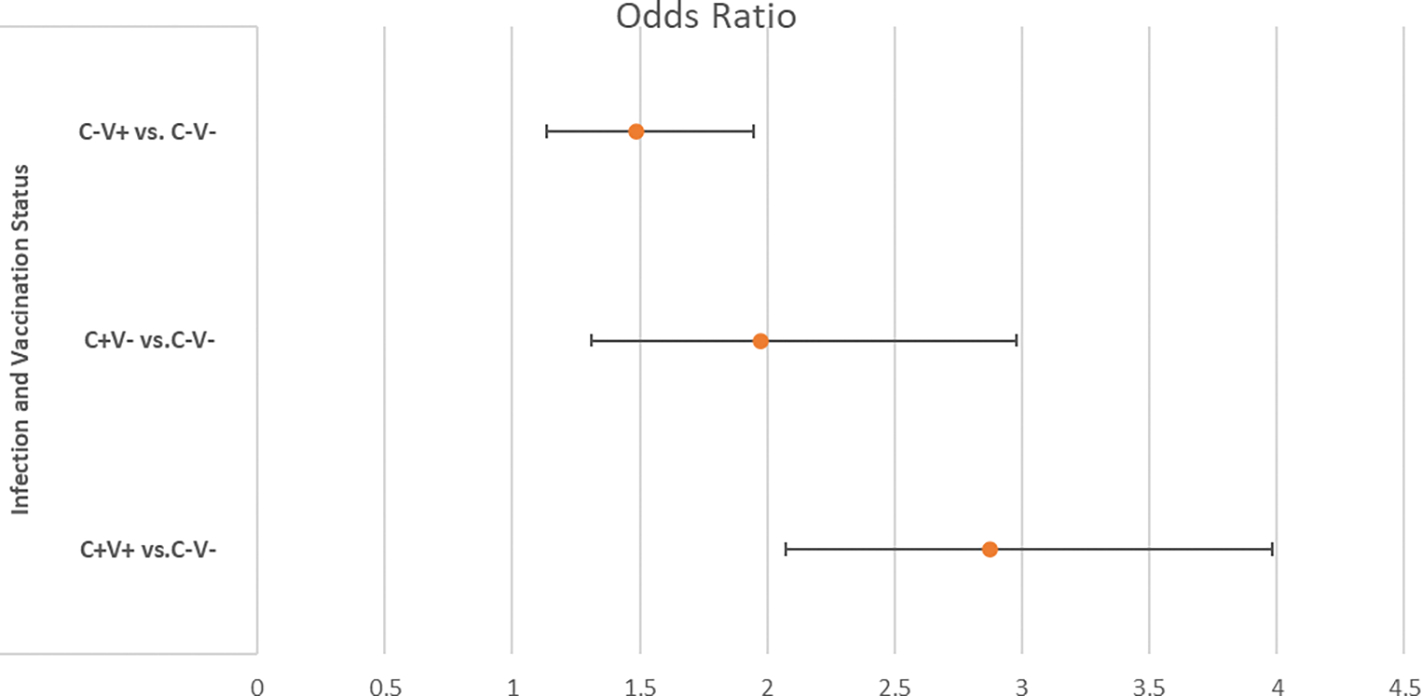
Odds (95%CI) of Develping IBS Among Different Groups of Veterans with “C−V−” Group as Reference. C−V−: no COVID infection and no vaccination; C−V+: no COVID infection, received vaccination; C+V−: COVID infection, no vaccination; C+V+: COVID infection, received vaccination.

In statistical terms, a lower p-value typically indicates stronger evidence of the null hypothesis. Therefore, the small p-values (0.004, 0.0012, and <0.0001) suggest that the differences in odds ratios for the C−V+, C+V−, and C+V+ groups compared to the C−V− reference group are statistically significant. This may imply a meaningful association or effect of IBS development in the populations represented by these groups (Table 2 and Figure 3).

Interestingly, if we chose C+V− as the reference group, the odds ratio of C+V+ with IBS was larger than one. However, the difference was not statistically significant, as indicated by a p-value of 0.084. The odds ratio for this association was 1.417, with a 95% confidence interval (CI) of 0.954–2.103. Meanwhile, the association of C−V+ with IBS was reduced compared with that of C+V−. Similarly, this reduction was not statistically significant, with a p-value of 0.1398. The odds ratio for this association was 0.761, with a 95% confidence interval (CI) of 0.530–1.093. The results of multivariate logistic regression analysis comparing the groups are presented in Table 3. In both cases, p-values were greater than the conventional significance level of 0.05, indicating that the observed associations were not statistically significant. This implies that the differences in the odds ratios may be due to chance, and there is no strong evidence to support a meaningful association between the groups and the presence of irritable bowel syndrome based on the given data.

## 4. Discussion

Although the relationship between COVID infection and IBS has been a topic of interest, available research on this topic that includes COVID vaccination status, is limited. It is important to note that the understanding of the long-term consequences of COVID-19, including its potential effects on various organ systems and the development of chronic conditions, is an evolving field of study. Studies that include information on the COVID vaccination status could provide valuable insights into the potential differences in the development of IBS between those who have been vaccinated and those who have not. At the onset of the pandemic, the primary focus of medical research was on the nature of the virus, its modes of transmission, and its impact on the respiratory system. Consequently, GI symptoms and IBS-like conditions have received little attention. Many early reports and case studies have indicated that some infected individuals experience GI symptoms including diarrhea, abdominal pain, nausea and vomiting, and changes in bowel habits. These symptoms are sometimes referred to as “gastrointestinal COVID”. However, these observations were largely anecdotal and based on a relatively small number of cases. Over time, larger reports have been published, but large epidemiological studies that include the effects of COVID vaccination on the development of IBS are lacking. This prompted us to conduct a population-based, cross-sectional study of veterans to better understand the relationship between COVID infection with or without COVID vaccination and the development of IBS.

Our current study demonstrates that veterans with COVID infection are almost twice as likely to develop IBS compared to those who have never had it. In theory, there are several potential mechanisms through which COVID infection could increase the risk of IBS development. One possible mechanism is that the infection may disrupt the gut microbiome, leading to dysbiosis. Dysbiosis has been suggested to be involved in the pathogenesis of IBS and COVID infection has been shown to alter the gut microbiome in both animal models and humans [17–19]. Another possible mechanism is that COVID may directly damage the intestinal epithelium, leading to increased intestinal permeability and enteric inflammation, which has been associated with IBS development [20–22]. A third possible mechanism is that COVID infection could induce an immune response that may persist even after recovery. Immune system dysregulation has been implicated in IBS [23, 24]. Another possible mechanism is that COVID infection and its associated consequences, such as quarantine, social isolation, and anxiety, can impact mental health in susceptible individuals. Psychosocial stress factors are known to influence gut function and can lead to the development or exacerbation of IBS symptoms, especially in veterans who have been in active military conflicts [25–30]. While one or more of these mechanisms are plausible, it is important to note that our current knowledge regarding the development of IBS is limited, and future research may reveal other yet unknown mechanisms.

The effect of COVID vaccination on IBS development seems to be more complex [31]. It can be influenced by various factors, including genetics, the gut microbiota, immune system function, and psychological factors. Vaccinations, including COVID-19 vaccines, primarily aim to stimulate the immune system to recognize and fight specific pathogens, and usually do not have a direct association with the development of IBS. Based on the present study, COVID vaccination may carry a small potential risk of IBS. Nevertheless, the study also indicates that individuals who received the vaccination and did not contract COVID infection had a lower risk of developing IBS compared to those who were unvaccinated and contracted the infection. In other words, vaccination, by preventing COVID infection—a factor associated with a heightened risk, may lead to an overall risk reduction if it proves highly effective in preventing infection. Understanding the complexities of these relationships would likely necessitate studies delving into immune responses, inflammatory pathways, and potential triggers for IBS in vaccinated individuals, both with and without COVID infection, offering an avenue for further research.

This study exhibits a nuanced interplay between strengths and weaknesses, underscoring its methodological intricacies. Among its noteworthy strengths is the utilization of a randomly selected sample, a hallmark of a robust research design aimed at mitigating bias. The adoption of random sampling strategies serves as a bulwark against selection bias, ensuring the equitable representation of all segments of the population under scrutiny. This approach not only bolsters the credibility of the study, but also augments the generalizability of its findings, extrapolating insights to the broader population from which the sample was drawn. Complementing this robust sampling technique is a commendable scale of the study, characterized by a relatively large sample size. A substantial cohort not only confers enhanced statistical power, facilitating the discernment of subtle effects or disparities with heightened precision but also fortifies the reliability and robustness of the study’s findings. Furthermore, the large sample size amplifies the external validity of the results, bolstering their applicability to diverse demographic strata.

However, these methodological strengths are a notable weakness: the inability to assess the duration or severity of COVID infection. These unmeasured variables are pivotal determinants that could potentially exert a significant influence on the risk of IBS development. Despite this limitation, the study yielded pivotal insights into the intricate interplay between COVID infection and the onset of IBS, shedding light on the direct association between the two phenomena. Moreover, the study offers tantalizing glimpses into the complex interrelationship between COVID vaccination and IBS development, hinting at multifaceted dynamics that warrant further exploration.

## 5. Conclusions

Our current study aimed to elucidate whether COVID infection confers a heightened risk for IBS development and to assess how vaccination against COVID influences this potential risk. While the study has certain methodological limitations, its substantive contributions to the existing body of knowledge are indisputable. Our current study demonstrates that veterans with COVID infection are almost twice as likely to develop IBS compared to those who have never had it. By unraveling the intricate nexus between COVID infection, vaccination, and the emergence of IBS, this study provides further insight into the enduring ramifications of COVID on gastrointestinal health. These findings underscore the need for sustained research aimed at elucidating the long-term repercussions of COVID infection and vaccination, particularly their intricate impact on the gastrointestinal milieu.

## Figures and Tables

**Table 1.** Baseline Characteristics of the Study Population.

	Total (n=9112)
Age (Mean ± SD)	65.34 ± 14.22
Sex	
Male	8019 (88.00%)
Female	1093 (12.00%)
Race	
While	3733 (40.97%)
Black	4931 (54.12%)
Other	448 (4.92%)
COVID Infection	
Yes	1651 (18.12%)
No	7461 (81.88%)
Vaccination Status	
Vaccinated	5038 (55.29%)
Not vaccinated	4074 (44.71%)
Vaccine Type	
Pfizer	4193 (83.23%)
Moderna	616 (12.23%)
Janssen	229 (4.55%)

**Table 2.** Effects of COVID Infection and Vaccination on Odds of IBS Development.

Group	Number (% of total population)	OR (95% CI)
C−V−	3413 (37.46%)	Reference group
C−V+	4048 (44.42%)	1.55 (1.19 – 2.02)
C+V−	661 (7.25%)	2.12 (1.41 – 3.18)
C+V+	990 (10.86%)	3.08 (2.24 – 4.24)

OR=Odds ratio; CI=Confidence interval, IBS=irritable bowel syndrome

**Table 3.** The risk ratios and 95% confidence intervals for development of IBS among the 4 groups of patients based on the combination of prior COVID-19 infection and vaccination status and after adjustment for age, gender, and racefactors.

		Univariate Model		Multivariate Model	
		Risk Ratio	p-value	Risk Ratio	p-value
Age	65+	1.170 (0.945,1.447)	0.1492	1.156 (0.926,1.445)	0.2011
	less than 65	Ref			
Sex	Male	0.921 (0.678,1.251)	0.5978	0.880 (0.639,1.211)	0.4318
	Female	Ref			
Race	White	Ref		Ref	
	Black	1.369 (1.100,1.704)	0.0049	1.205 (0.963,1.508)	0.1037
	Other	0.833 (0.464,1.496)	0.5413	0.801 (0.446,1.439)	0.458
Covid Vaccine	C−V+ Vs C−V−	1.529 (1.180, 1.982)	0.0013	1.469 (1.131, 1.909)	0.0040
	C+V− Vs C−V−	2.041 (1.385,3.010)	0.0003	1.930 (1.304, 2.856)	0.0012
	C+V+ Vs C−V−	2.926 (2.160,3.965)	<0.0001	2.706 (1.983, 3.692)	<0.0001
	C−V+ Vs C+V−	0.749 (0.522,1.076)	0.1177	0.761 (0.530, 1.093)	0.1398
	C+V+ Vs C+V−	1.434 (0.966, 2.128)	0.0738	1.417 (0.954, 2.103)	0.0840

## Abbreviations

CI: Confidence Interval
COVID: Coronavirus Disease
IBS: Irritable Bowel Syndrome
OR: Odds Ratio
PCR: Polymerase Chain Reaction
VA: Veterans Affairs
